# The Relationship Between Social Determinants of Health and Cigarette Smoking Behaviors Among Adults in the United States, Behavioral Risk Factor Surveillance System (BRFSS), 2023

**DOI:** 10.3390/ijerph23030292

**Published:** 2026-02-27

**Authors:** Sabrina L. Smiley, Molly Hendricks, Heesung Shin

**Affiliations:** Division of Health Promotion and Behavioral Science, School of Public Health, San Diego State University, San Diego, CA 92182-8080, USA; mhendricks9346@sdsu.edu (M.H.); hshin4@sdsu.edu (H.S.)

**Keywords:** social determinants, cigarette smoking, cessation, menthol

## Abstract

**Highlights:**

**Public health relevance—How does this work relate to a public health issue?**

- Social determinants of health (SDoH) contribute to the excess risk of cigarette smoking.

**Public health significance—Why is this work of significance to public health?**

- This study uses a large, nationally representative dataset to examine associations between SDoH and cigarette smoking-related outcomes (i.e., cigarette smoking status, menthol cigarette smoking, and past-year quit attempt) among U.S. adults.
- Individual SDoH risk factors (i.e., food insecurity, housing insecurity, utility insecurity, and lack of reliable transportation) were significantly associated with making a quit attempt in the past 12 months. Food insecurity and utility insecurity were independent risk factors for using menthol cigarettes.

**Public health implications—What are the key implications or messages for practitioners, policymakers, and/or researchers in public health?**

- Interventions and screenings aimed at reducing SDoH risk factors (i.e., food insecurity and utility insecurity) may help reduce the prevalence of cigarette smoking, including menthol cigarette smoking, and increase the prevalence of smoking cessation among U.S. adults.

**Abstract:**

Social determinants of health (SDoH) comprise a broad array of social conditions, such as access to food and housing, that facilitate or impede individual behavior. The aim of this study was to assess the association between SDoH and cigarette smoking-related outcomes among U.S. adults (aged ≥18 years) by using data from the 2023 Behavioral Risk Factor Surveillance System (BRFSS). Cross-sectional data were obtained from the Social Determinants and Health Equity (SD/HE) module, conducted in 33 states, the District of Columbia, and Puerto Rico as part of the 2023 BRFSS. We examined four indicators of adverse SDoH (i.e., food insecurity, housing insecurity, utility insecurity, and lack of reliable transportation) and three cigarette smoking-related outcomes (i.e., cigarette smoking status, menthol cigarette smoking, and past-year quit attempt). All analyses were conducted with SAS 9.4 and used BRFSS sampling weights to adjust for the complex sampling design. Among 45,160 respondents, 2991 (7.8%) were adults who smoked cigarettes in the past month, of whom 570 (16.5%) reported making a quit attempt in the past 12 months. Menthol cigarette use was reported by 634 (22.0%) adults who smoked cigarettes in the past month. In adjusted analyses, each SDoH measure (i.e., food insecurity (aOR = 1.70, 95% CI: 1.19–2.41, *p* < 0.01), housing insecurity (aOR = 1.66, 95% CI: 1.06–2.59, *p* < 0.05), utility insecurity (aOR = 1.92, 95% CI: 1.01–3.65, *p* < 0.05), and lack of reliable transportation (aOR = 1.67, 95% CI: 1.03–2.73, *p* < 0.05)) was significantly associated with making a quit attempt in the past 12 months. Food insecurity was significantly associated with the odds of current cigarette smoking. Food insecurity and utility insecurity were independent risk factors for using menthol cigarettes. U.S. adults experiencing adverse SDoH are trying to stop smoking at higher rates than adults not experiencing adverse SDoH. Findings demonstrate that SDoH is a strong predictor of cigarette smoking status, menthol cigarette smoking, and past-year quit attempts among U.S. adults.

## 1. Introduction

Although current (past 30 days) cigarette smoking among U.S. adults overall has steadily declined over the past few decades [1], the progress made has not been equal for everyone [2]. Disparities in cigarette smoking persist by racial and ethnic group, educational attainment, and menthol cigarette smoking status, among other factors [2,3,4,5]. In 2023, current smoking prevalence was 18.3% among adults with less than a high school education, compared with 4.4% among adults with an undergraduate college degree [6]. Cigarette smoking remains the leading cause of preventable disease and death in the U.S., accounting for approximately 500,000 or one in five deaths each year [7]. Accelerated efforts to prevent and treat cigarette smoking for those who bear the greatest burden could have significant public health and clinical impacts.

Many factors influence an individual’s smoking behavior, including social determinants of health (SDoH), defined as social conditions in which individuals grow, live, and work that affect their exposure to health-related risks [8]. SDoH comprise a broad array of social conditions, such as access to food and housing. In a nationally representative sample of U.S. adults, higher odds of making quit attempts in the past 12 months were associated with current smokers who identified as non-Hispanic Black, graduated from college, and reported food and housing insecurity [9]. For instance, combustible tobacco use can exacerbate food insecurity, defined as having limited or uncertain access to adequate food due to limited economic resources [10]. Purchasing cigarettes may divert financial resources from sufficient food access, disproportionately impacting low-income households [11]. SDoH can also facilitate or hinder smoking cessation efforts. Treatment of tobacco dependence is critical to reduce the persistent disparities in cigarette use and subsequent adverse health outcomes.

Therefore, the purpose of this study was to provide nationally representative data on SDoH and cigarette smoking behaviors. Specifically, this study assessed the association between four indicators of adverse SDoH (i.e., food insecurity, housing insecurity, utility insecurity, and lack of reliable transportation) and three cigarette smoking-related outcomes (i.e., cigarette smoking status, menthol cigarette smoking, and past-year quit attempt), using data from the 2023 Behavioral Risk Factor Surveillance System (BRFSS). Considering the health-related risks associated with smoking and the disparities that persist in smoking behaviors in adults, examining predictors of use within this population provides important insights to inform intervention development.

## 2. Methods

### 2.1. Data Source

This cross-sectional study used data from the 2023 Behavioral Risk Factor Surveillance System (BRFSS), a public data survey conducted by the Centers for Disease Control and Prevention to collect information, including sociodemographics and health-related risk behaviors [12]. The BRFSS is an annual phone-based survey conducted in all 50 states, the District of Columbia, and U.S. territories (Guam, Puerto Rico, and the U.S. Virgin Islands) that includes non-institutionalized U.S. residents 18 years of age and older [12]. For the 2023 BRFSS, the median response rate was 44.6%, ranging from 21.7% in Guam to 63.1% in Alaska. The BRFSS utilizes a complex multistage cluster sampling design to adjust for non-response and selection bias, and a weighting method called iterative proportional fitting, or raking, was used to weight the data [12]. A total of 33 states, the District of Columbia (DC), and Puerto Rico used the optional Social Determinants and Health Equity module in the 2023 BRFSS. The BRFSS is a publicly available dataset containing no identifying participant information and thus does not meet the requirements of human subjects research by the San Diego State University IRB.

### 2.2. Measures

Current cigarette smoking was assessed by two questions: “Have you smoked at least 100 cigarettes in your entire life?” (no, yes) and “Do you now smoke cigarettes every day (no, yes), some days (no, yes), or not at all (no, yes)?”Adults who currently smoke cigarettes were defined as respondents ≥age 18 who have smoked at least 100 cigarettes in their lifetime and currently smoke on at least some days. Menthol cigarette smoking was defined as answering yes to the following question: “Currently, when you smoke cigarettes, do you usually smoke menthol cigarettes?” Making a past-year quit attempt was defined as answering yes to the following question: “During the past 12 months, have you stopped smoking for one day or longer because you were trying to quit smoking?”.

SDoH measures included food insecurity, housing insecurity, utility insecurity, and lack of reliable transportation. To assess food insecurity, responses (never, rarely, sometimes, usually, always) were analyzed for the question “During the past 12 months how often did the food that you bought not last, and you didn’t have money to get more?” To assess housing insecurity, responses (no, yes) were analyzed for the question, “During the last 12 months, was there a time when you were not able to pay your mortgage, rent, or utility bills?” To assess utility insecurity, responses (no, yes) were analyzed for the following question: “During the last 12 months was there a time when an electric, gas, oil, or water company threatened to shut off services?” To assess lack of reliable transportation, responses (no, yes) were analyzed for the following question: “During the past 12 months, has a lack of reliable transportation kept you from medical appointments, meetings, work, or from getting things needed for daily living?”.

The following factors were examined as covariates: age (18–24, 25–34, 35–44, 45–54, 55–64, and ≥65 years); sex (male and female); race and ethnicity (Hispanic, non-Hispanic American Indian or Alaska Native, non-Hispanic Asian, non-Hispanic Black or African American, non-Hispanic White, and non-Hispanic Other race); educational attainment (did not graduate high school, graduated high school, attended college or technical school, and graduated college or technical school); marital status (married, previously married [divorced, widowed, or separated], never married, or living with a partner); annual household income (<$15,000, $15,000 to <$25,000, $25,000 to <$35,000, $35,000 to <$50,000, $50,000 to <$100,000, $100,000 to <$200,000, and ≥$200,000); health insurance coverage (no, yes); and general health rated as fair or poor, number of days physical health was not good for more than 15 days/month, and number of days mental health was not good for more than 15 days/month. To ensure analytic stability, adequate cell sizes, and interpretability, educational attainment was recoded to 1 = some college versus 0 = not; household income to 1 = ≥$50,000 versus 0 = <$50,000; and marital status to 1 = married versus 0 = not.

### 2.3. Statistical Analysis

All statistical analyses were performed utilizing SAS 9.4 (SAS Institute Incorporated, Cary, NC, USA) in July 2025. Because the BRFSS employs complex survey designs, SAS survey procedures (e.g., SURVEYFREQ and SURVEYLOGISTIC) were used to account for sampling weights, clustering, and stratification. Multivariable logistic regression models were used to examine associations between each smoking-related outcome and the SDoH measure, adjusting for age, sex, race and ethnicity, level of education completed, marital status, annual household income, health insurance coverage, and general health. Crude and adjusted odds ratios and their corresponding 95% confidence intervals (CIs) were calculated to indicate the strength of the association between each outcome and the SDoH measure. All models were adjusted for age, sex, race and ethnicity, level of education completed, marital status, annual household income, health insurance coverage, and general health, with SDoH indicators (food insecurity, housing insecurity, utility insecurity, and lack of reliable transportation) included simultaneously. Statistical significance of estimates was determined at the 5% level.

## 3. Results

Sample (*N* = 45,160) characteristics and key study variables are summarized in Table 1. Overall, the majority of respondents were aged 55 years and older (82.8%), female (62.4%), and non-Hispanic White (73.99%). Less than 10% did not graduate high school, and 29.35% had graduated from college or technical school. More than half (58.50%) were married. An annual household income below $50,000 accounted for 36.4% of respondents; 41.1% had an annual household income above $50,000. Approximately one-fifth (22.49%) provided no information on income.

Current/past month cigarette smoking was reported by 2991 (6.6%) respondents, and 570 (19.1%) who smoked cigarettes in the past month reported making a quit attempt in the past 12 months. Menthol cigarette use was reported by 634 (21.2%) respondents who smoked cigarettes in the past month. Respondents who reported menthol cigarette use were significantly more likely than their counterparts to identify as non-Hispanic Black and make a quit attempt in the past 12 months. Compared with adults who did not report current cigarette use, those who reported current use were significantly (*p* < 0.001) more likely to experience food insecurity (20.1% versus 8.7%), lack of reliable transportation (11.8% versus 4.9%), housing insecurity (10.2% versus 5.35%), and utility insecurity (7.1% versus 3.5%). Most (97.5%) had health insurance, and 16.58% reported their physical health was not good for more than 15 days/month.

In adjusted analyses (Table 2), each SDoH measure (i.e., food insecurity, housing insecurity, utility insecurity, and lack of reliable transportation) was significantly associated with making a quit attempt in the past 12 months (aOR = 1.70, 95% CI: 1.19–2.41, *p* < 0.01; aOR = 1.66, 95% CI: 1.06–2.59, *p* < 0.05; aOR = 1.92, 95% CI: 1.01–3.65, *p* < 0.05; aOR = 1.67, 95% CI: 1.03–2.73, *p* < 0.05). Food insecurity was significantly associated with the odds of current cigarette smoking (aOR = 1.63, 95% CI: 1.21–2.20, *p* < 0.001). Food insecurity and utility insecurity were significantly associated with the odds of menthol cigarette smoking (aOR = 1.58, 95% CI: 0.99–2.51, *p* < 0.05; aOR = 1.99, 95% CI: 1.11–3.58, *p* < 0.05). In addition, higher education, higher income, and being married were significantly associated with lower odds of cigarette smoking (aOR = 0.39, 95% CI: 0.31–0.48, *p* < 0.001; aOR = 0.80, 95% CI: 0.64–0.99, *p* < 0.05; aOR = 0.61, 95% CI: 0.51–0.73, *p* < 0.001); menthol cigarette smoking (aOR = 0.48, 95% CI: 0.32–0.72, *p* < 0.001; aOR = 0.58, 95% CI: 0.36–0.93, *p* < 0.01; aOR = 0.52, 95% CI: 0.34–0.81, *p* < 0.01); and making a quit attempt in the past 12 months (aOR = 0.45, 95% CI: 0.31–0.65, *p* < 0.001; aOR = 0.60, 95% CI: 0.47–0.77, *p* < 0.001; aOR = 0.78, 95% CI: 0.61–1.00, *p* < 0.10). Further, experiencing 15 or more mentally unhealthy days during the past 30 days was significantly associated with the odds of current cigarette smoking (aOR = 1.44, 95% CI: 1.11–1.86, *p* < 0.01).

## 4. Discussion

This cross-sectional study examined the relationship between adverse SDoH, cigarette smoking, and quit attempts in a national U.S. sample of adults. We found that each SDoH measure (i.e., food insecurity, housing insecurity, utility insecurity, and lack of reliable transportation) was significantly associated with making a quit attempt in the past 12 months. Additionally, food insecurity was significantly associated with cigarette smoking, including menthol cigarette smoking. Given that less than 10% of adults who smoke cigarettes succeed in quitting in a given year [13], efforts to reduce cigarette smoking and increase cessation should address experiences of SDoH, including food insecurity. Further research is needed to investigate the underlying mechanisms that may contribute to the observed associations between SDoH, cigarette smoking, and making a quit attempt in the past 12 months.

In the study sample, 6.6% of U.S. adults reported current cigarette smoking, and 21% of them reported current menthol cigarette smoking. Despite progress in reducing cigarette smoking in the U.S. population and among certain demographic groups [14], non-Hispanic Black adults were associated with increased odds of reporting current menthol cigarette smoking. Consistent with prior studies [9,15], food insecurity was significantly associated with current smoking. This is also among the first studies to highlight that food insecurity and utility insecurity are independent risk factors for menthol cigarette smoking. Better understanding the potential reciprocal nature and mechanisms is needed to inform efforts to improve cessation outcomes. Furthermore, combustible tobacco use policies, such as a federal ban on menthol cigarettes, are needed, as they may reduce food insecurity and utility insecurity among U.S. adults who smoke [16,17].

Most people who smoke cigarettes make repeated attempts to stop smoking before achieving sustained abstinence [18,19]. Understanding SDoH as a predictor of making a quit attempt in the past 12 months may help guide interventions, programming efforts, and messaging to increase smoking cessation at the population level. Public health practitioners who tailor efforts to people who smoke cigarettes may want to consider the role of SDoH in supporting and inhibiting a quit attempt. For example, interventions aimed at increasing smoking cessation in adults may want to focus on SDoH, as our study found it was significantly associated with making a quit attempt in the past 12 months. Because individuals experiencing adverse SDoH have worse health outcomes [20], SDoH-informed smoking cessation interventions are necessary.

### Limitations

The current study is not without limitations. First, BRFSS data are self-reported, which can lead to underreporting and misclassification bias. Second, we cannot examine whether the associations identified are causal because BRFSS is a cross-sectional survey and cannot establish temporality. Longitudinal research is needed to clarify such directions. Third, respondents were primarily non-Hispanic White, aged 55 years and older, and had health insurance, which means findings may not be generalizable to other demographic groups. Finally, states that did not participate in the optional module estimating SDoH conditions were excluded, so generalizability of the study results to the U.S. population is limited. Despite these limitations, the strengths of this study include using a large population-based sample. Additionally, this study provides invaluable data to facilitate targeted SDoH-informed smoking cessation interventions.

## 5. Conclusions

U.S. adults experiencing adverse SDoH are trying to stop smoking at higher rates than adults not experiencing adverse SDoH. Findings demonstrate that adverse SDoH is a strong predictor of cigarette smoking status, menthol cigarette smoking, and making a quit attempt in the past 12 months among U.S. adults. Findings support clinician screening for SDoH and cigarette smoking in adults and providing treatment or referrals when needed. Furthermore, these findings can be used to guide tailored educational messaging for smoking-related harms and the benefits of quitting. Messaging can focus on the SDoH (e.g., food insecurity) related to smoking behaviors.

## Figures and Tables

**Table 1:** Characteristics of study participants (*N* = 45,160), BRFSS 2023, United States.

	Weighted % ^a^ (95% CI)	Current (Past 30 Days) Cigarette Smoking Weighted % ^a^ (95% CI)			Current (Past 30 Days) Menthol Cigarette Smoking Weighted % ^a^ (95% CI)			Past 12 Months Quit Attempt Weighted % ^a^ (95% CI)		
Characteristic	Overall (***N*** = 45,160)	No (***N*** = 42,169, 93.38%)	Yes (***N*** = 2991, 6.62%)	***p*** Value	No (***N*** = 2357, 78.80%)	Yes (***N*** = 634, 21.20%)	***p*** Value	No (***N*** = 2421, 80.94%)	Yes (***N*** = 570, 19.06%)	***p*** Value
Age (Years)										
18–24	0.98% (0.66, 1.31)	1.00% (0.67, 1.34)	0.74% (0.00, 2.04)	<0.001	0.95% (0.00, 2.61)		-	0.89% (0.00, 2.44)		-
25–34	1.80% (1.41, 2.18)	1.82% (1.41, 2.23)	1.49% (0.67, 2.31)		1.38% (0.41, 2.35)	1.89% (0.46, 3.32)		1.57% (0.61, 2.54)	1.08% (0.06, 2.10)	
35–44	4.16% (3.62, 4.71)	4.04% (3.46, 4.61)	5.61% (3.94, 7.29)		5.58% (3.70, 7.46)	5.74% (2.09, 9.38)		5.46% (3.61, 7.30)	6.41% (2.50, 10.31)	
45–54	10.22% (9.45, 10.99)	10.02% (9.22, 10.81)	12.60% (9.76, 15.44)		13.42% (10.02, 16.83)	9.68% (5.15, 14.20)		12.59% (9.37, 15.80)	12.66% (6.93, 18.39)	
55–64	19.56% (18.59, 20.53)	18.54% (17.57, 19.50)	31.65% (27.13, 36.16)		31.87% (26.58, 37.15)	30.87% (22.47, 39.27)		31.21% (26.00, 36.43)	33.85% (26.35, 41.35)	
≥65	63.28% (62.12, 64.43)	64.58% (63.39, 65.77)	47.91% (43.67, 52.14)		46.80% (41.96, 51.64)	51.83% (43.38, 60.28)		48.28% (43.42, 53.15)	46.01% (38.68, 53.33)	
Sex										
Female	62.39% (61.31, 63.46)	62.36% (61.24, 63.48)	62.71% (58.57, 66.84)	0.87	62.54% (57.74, 67.33)	63.31% (55.27, 71.36)	0.87	62.74% (58.03, 67.45)	62.55% (54.77, 70.33)	0.97
Male	37.61% (36.54, 38.69)	37.64% (36.52, 38.76)	37.29% (33.16, 41.43)		37.46% (32.67, 42.26)	36.69% (28.64, 44.73)		37.26% (32.55, 41.97)	37.45% (29.67, 45.23)	
Race and Ethnicity										
Hispanic	7.31% (6.44, 8.18)	7.62% (6.69, 8.55)	3.60% (1.88, 5.33)		3.37% (1.67, 5.06)	4.43% (0.00, 9.44)		4.05% (2.00, 6.10)	1.33% (0.13, 2.53)	
Non-Hispanic American Indian or Alaska Native	0.89% (0.74, 1.04)	0.87% (0.71, 1.02)	1.21% (0.77, 1.66)		1.15% (0.67, 1.63)	1.44% (0.33, 2.55)		1.31% (0.79, 1.83)	0.73% (0.20, 1.26)	
Non-Hispanic Asian	3.48% (2.72, 4.24)	3.73% (2.91, 4.56)	0.53% (0.16, 0.90)		0.27% (0.01, 0.54)	1.42% (0.04, 2.81)		0.61% (0.17, 1.05)	0.09% (0.00, 0.18)	
Non-Hispanic Black	11.54% (10.83, 12.25)	11.68% (10.95, 12.41)	9.90% (6.84, 12.96)		7.08% (3.64, 10.52)	19.87% (13.12, 26.63)		9.68% (6.20, 13.16)	11.00% (5.17, 16.83)	
Non-Hispanic White	73.99% (72.80, 75.17)	73.32% (72.07, 74.56)	81.87% (78.36, 85.39)	<0.001	84.93% (81.02, 88.83)	71.09% (63.26, 78.91)	<0.001	81.19% (77.15, 85.23)	85.34% (79.36, 91.32)	0.56
Other, Non-Hispanic	2.79% (2.40, 3.18)	2.78% (2.37, 3.19)	2.88% (1.72, 4.05)		3.20% (1.76, 4.65)	1.75% (0.40, 3.10)		3.16% (1.79, 4.52)	1.51% (0.13, 2.89)	
Level of Education Completed										
Did not Graduate High School	9.74% (8.80, 10.68)	9.31% (8.33, 10.29)	14.74% (11.26, 18.22)	<0.001	15.31% (11.40, 19.22)	12.73% (5.00, 20.45)	0.42	13.34% (9.42, 17.25)	21.83% (14.61, 29.05)	<0.10
Graduated High School	28.15% (27.21, 29.10)	27.56% (26.58, 28.54)	35.12% (31.39, 38.85)		34.13% (30.00, 38.27)	38.60% (30.35, 46.86)		34.92% (30.66, 39.18)	36.14% (29.38, 42.90)	
Attended College or Technical School	32.76% (31.69, 33.83)	32.29% (31.20, 33.39)	38.26% (33.83, 42.70)		39.52% (34.27, 44.77)	33.81% (26.64, 40.99)		39.61% (34.50, 44.71)	31.46% (24.75, 38.17)	
Graduated from College or Technical School	29.35% (28.42, 30.28)	30.83% (29.84, 31.82)	11.88% (9.75, 14.01)		11.03% (8.85, 13.22)	14.86% (9.15, 20.57)		12.14% (9.80, 14.48)	10.57% (5.32, 15.81)	
Marital Status										
Married	58.50% (57.39, 59.61)	59.88% (58.74, 61.02)	42.19% (38.18, 46.21)	<0.001	43.45% (38.77, 48.14)	37.73% (29.96, 45.50)	0.33	41.75% (37.18, 46.33)	44.41% (37.05, 51.78)	0.75
Divorced	9.76% (9.16, 10.37)	9.01% (8.42, 9.59)	18.66% (15.31, 22.01)		19.49% (15.52, 23.46)	15.71% (9.95, 21.47)		18.31% (14.46, 22.17)	20.40% (14.67, 26.12)	
Widowed	19.10% (18.20, 20.01)	19.13% (18.18, 20.07)	18.83% (15.77, 21.90)		17.47% (14.42, 20.52)	23.65% (15.28, 32.02)		19.51% (15.92, 23.09)	15.42% (11.51, 19.33)	
Separated	1.04% (0.77, 1.30)	0.88% (0.62, 1.15)	2.81% (1.46, 4.17)		3.16% (1.47, 4.86)	1.58% (0.14, 3.02)		2.70% (1.20, 4.19)	3.41% (0.16, 6.66)	
Never married	9.96% (9.23, 10.70)	9.62% (8.92, 10.31)	14.03% (9.60, 18.46)		13.23% (7.77, 18.69)	16.88% (10.85, 22.90)		14.53% (9.38, 19.67)	11.54% (5.37, 17.71)	
A Member of an Unmarried Couple	1.64% (1.35, 1.93)	1.48% (1.20, 1.77)	3.47% (1.86, 5.08)		3.20% (1.46, 4.93)	4.45% (0.50, 8.40)		3.21% (1.54, 4.87)	4.82% (0.00, 9.77)	
Employment Status										
Employed for Wages	21.38% (20.46, 22.31)	21.55% (20.57, 22.52)	19.43% (16.60, 22.27)	<0.001	19.24% (16.01, 22.48)	20.10% (14.23, 25.96)	0.74	20.25% (16.96, 23.54)	15.28% (10.75, 19.81)	<0.05
Self-employed	5.54% (5.07, 6.01)	5.50% (5.02, 5.97)	6.05% (3.90, 8.20)		6.27% (3.68, 8.87)	5.24% (1.92, 8.57)		5.69% (3.31, 8.07)	7.86% (2.88, 12.83)	
Out of Work for 1 Year or More	1.46% (1.11, 1.81)	1.30% (0.94, 1.66)	3.32% (2.00, 4.63)		3.05% (1.78, 4.33)	4.25% (0.35, 8.15)		2.66% (1.46, 3.87)	6.62% (1.61, 11.64)	
Out of Work for Less Than 1 Year	0.90% (0.71, 1.10)	0.80% (0.61, 0.99)	2.16% (1.04, 3.27)		2.21% (0.84, 3.57)	1.99% (0.41, 3.56)		2.29% (0.99, 3.59)	1.49% (0.00, 3.08)	
A Homemaker	6.01% (5.41, 6.60)	6.16% (5.53, 6.80)	4.17% (3.05, 5.28)		4.55% (3.19, 5.92)	2.80% (1.28, 4.32)		4.22% (2.95, 5.50)	3.88% (1.93, 5.84)	
A Student	0.91% (0.56, 1.26)	0.96% (0.58, 1.34)	0.28% (0.00, 0.66)		0.13% (0.00, 0.33)	0.83% (0.00, 2.39)		0.13% (0.00, 0.32)	1.07% (0.00, 3.15)	
Retired	56.21% (55.08, 57.35)	57.06% (55.89, 58.24)	46.22% (41.99, 50.46)		45.76% (40.90, 50.62)	47.87% (39.42, 56.32)		47.47% (42.59, 52.35)	39.92% (32.92, 46.92)	
Unable to Work	7.59% (6.86, 8.32)	6.67% (5.99, 7.35)	18.38% (13.93, 22.82)		18.79% (13.46, 24.12)	16.92% (9.89, 23.94)		17.29% (12.12, 22.46)	23.88% (16.79, 30.96)	
Annual Household Income, $										
<$15,000	3.92% (3.38, 4.47)	3.66% (3.09, 4.23)	7.05% (5.41, 8.70)	<0.001	7.24% (5.32, 9.17)	6.38% (3.34, 9.42)	0.77	5.95% (4.28, 7.62)	12.64% (7.64, 17.64)	<0.01
$15,000 to <$25,000	9.12% (8.36, 9.88)	8.60% (7.81, 9.38)	15.29% (12.35, 18.24)		14.39% (11.22, 17.55)	18.49% (11.34, 25.65)		14.75% (11.43, 18.06)	18.05% (11.98, 24.13)	
$25,000 to <$35,000	10.83% (10.11, 11.55)	10.38% (9.71, 11.06)	16.11% (11.72, 20.51)		15.93% (10.59, 21.26)	16.78% (10.25, 23.31)		16.80% (11.64, 21.97)	12.63% (8.26, 17.00)	
$35,000 to <$50,000	12.57% (11.88, 13.25)	12.54% (11.82, 13.25)	12.93% (10.62, 15.23)		12.85% (10.29, 15.42)	13.18% (8.01, 18.36)		12.32% (9.91, 14.73)	15.99% (9.44, 22.54)	
$50,000 to <$100,000	23.29% (22.44, 24.14)	23.58% (22.69, 24.47)	19.84% (17.18, 22.51)		19.46% (16.48, 22.44)	21.18% (15.28, 27.07)		19.69% (16.71, 22.66)	20.62% (14.84, 26.40)	
$100,000 to <$200,000	13.80% (12.98, 14.62)	14.41% (13.53, 15.28)	6.61% (4.96, 8.26)		7.17% (5.20, 9.13)	4.63% (1.86, 7.39)		6.92% (5.02, 8.81)	5.04% (2.14, 7.94)	
$200,000 or more	3.98% (3.56, 4.39)	4.18% (3.75, 4.61)	1.61% (0.02, 3.20)		1.86% (0.00, 3.88)	0.73% (0.00, 1.71)		1.93% (0.02, 3.83)	0.01% (0.00, 0.02)	
Don’t Know/Not Sure/Missing	22.49% (21.56, 23.43)	22.66% (21.70, 23.62)	20.56% (16.89, 24.22)		21.10% (16.93, 25.27)	18.62% (10.88, 26.36)		21.65% (17.38, 25.92)	15.02% (10.23, 19.82)	
Cigarette Smoking Status										
Current (Past 30 Days) Smoking	7.83% (7.19, 8.47)	-	-		100.00% (100, 100)	100.00% (100, 100)		100.00% (100, 100)	100.00% (100, 100)	<0.001
Menthol Cigarette Smoking	2.69% (2.08, 3.31)	-	22.04% (18.68, 25.40)		0%	100.00% (100, 100)		19.95% (16.29, 23.60)	32.65% (24.96, 40.34)	<0.01
Past 12 Months Quit Attempt	2.17% (1.79, 2.55)	-	16.49% (14.02, 18.97)		14.25% (11.81, 16.69)	24.43% (17.65, 31.21)	<0.01	0	100	
Social Determinant of Health										
Food Insecurity	9.55% (8.82, 10.28)	8.66% (7.93, 9.38)	20.07% (16.43, 23.70)	<0.0001	18.56% (14.59, 22.53)	25.39% (16.98, 33.81)	0.12	19.10% (14.97, 23.24)	24.94% (18.01, 31.88)	0.14
Housing Insecurity	5.64% (5.02, 6.27)	5.25% (4.60, 5.91)	10.24% (8.01, 12.46)	<0.0001	9.79% (7.37, 12.22)	11.80% (6.49, 17.11)	0.48	8.47% (6.27, 10.67)	19.16% (12.16, 26.15)	<0.001
Lack of Reliable Transportation	5.47% (4.87, 6.08)	4.94% (4.34, 5.54)	11.75% (8.66, 14.84)	<0.0001	12.22% (8.46, 15.97)	10.11% (5.69, 14.52)	0.48	11.25% (7.69, 14.80)	14.30% (8.92, 19.67)	0.34
Utility Insecurity	3.74% (3.25, 4.24)	3.46% (2.94, 3.97)	7.12% (5.40, 8.84)	<0.0001	6.63% (4.86, 8.39)	8.86% (4.24, 13.49)	0.33	6.42% (4.69, 8.16)	10.64% (5.24, 16.04)	<0.10
General Health Rating										
General Health Rated as Fair/Poor	23.84% (22.87, 24.81)	22.84% (21.84, 23.84)	35.62% (31.70, 39.53)	<0.0001	34.95% (30.63, 39.28)	37.96% (29.17, 46.76)	0.54	33.43% (29.05, 37.82)	46.67% (39.15, 54.20)	<0.05
15+ Days/Month of Poor Physical Health	16.58% (15.66, 17.49)	15.64% (14.74, 16.54)	27.58% (23.01, 32.14)	<0.0001	17.90% (14.77, 21.03)	25.68% (16.37, 34.99)	0.65	18.54% (14.90, 22.18)	25.05% (17.81, 32.29)	<0.01
15+ Days/Month of Poor Mental Health	10.01% (9.39, 10.64)	9.20% (8.59, 9.81)	19.61% (16.33, 22.89)	<0.0001	27.05% (21.76, 32.34)	29.43% (20.45, 38.40)	<0.10	26.68% (21.37, 31.99)	32.10% (24.82, 39.38)	<0.001
Health Insurance										
Yes	97.51% (97.08, 97.94)	97.66% (97.22, 98.11)	95.74% (94.01, 97.47)	<0.01	95.64% (93.58, 97.70)	96.09% (93.14, 99.04)	0.81	95.85% (93.91, 97.79)	95.17% (91.50, 98.84)	0.74
No	2.49% (2.06, 2.92)	2.34% (1.89, 2.78)	4.26% (2.53, 5.99)		4.36% (2.30, 6.42)	3.91% (0.96, 6.86)		4.15% (2.21, 6.09)	4.83% (1.16, 8.50)	

Abbreviations: BRFSS, Behavioral Risk Factor Surveillance System. ^a^ Percentages may not add to 100 because of rounding.

**Table 2:** Adjusted associations between social determinants of health, selected covariates, and cigarette smoking-related behaviors, BRFSS 2023, United States.

		Current (Past 30 Days) Cigarette Smoking (*N* = 2991)	Current (Past 30 Days) Menthol Cigarette Smoking (*N* = 634)	Past 12 Months Quit Attempt (*N* = 570)
Sociodemographics				
Age groupsc ^a^		0.82 (0.75–0.89) ***	0.95 (0.84–1.07)	0.90 (0.81–0.99) *
Sex (Male = 1)		1.08 (0.89–1.31)	1.43 (0.89–2.30)	1.13 (0.85–1.51)
Race/Ethnicity (White, NH = 1) vs. Other Race/Ethnicity, Non-Hispanic		1.21 (0.76–1.94)	2.19 (1.04–4.65) *	2.54 (1.16–5.55) *
Race/Ethnicity (Black, NH = 1) vs. Other Race/Ethnicity, Non-Hispanic		0.71 (0.40–1.25)	3.48 (1.65–7.34) **	1.90 (0.81–4.43)
Race/Ethnicity (Asian = 1, NH) vs. Other Race/Ethnicity, Non-Hispanic		0.24 (0.10–0.57) **	1.11 (0.34–3.65)	0.15 (0.04–0.58) **
Race/Ethnicity (American Indian/Alaskan Native = 1) vs. Other Race/Ethnicity, Non-Hispanic		1.00 (0.54–1.85)	2.48 (0.95–6.51) +	1.28 (0.50–3.29)
Race/Ethnicity (Hispanic = 1) vs. Other Race/Ethnicity, Non-Hispanic		0.38 (0.19–0.77) **	2.77 (0.85–9.06) +	1.92 (0.45–8.20)
Level of Education Completed (Some College = 1) vs. Others ^b^		0.39 (0.31–0.48) ***	0.48 (0.32–0.72) ***	0.45 (0.31–0.65) ***
Annual Household Income (<$50,000 = 1) vs. Others ^c^		0.80 (0.64–0.99) *	0.58 (0.36–0.93) *	0.60 (0.47–0.77) ***
Married		0.61 (0.51–0.73) ***	0.52 (0.34–0.81) **	0.78 (0.61–1.00) +
Social Determinants of Health				
Food Insecurity		1.63 (1.21–2.20) **	1.58 (0.99–2.51) *	1.70 (1.19–2.41) **
Housing Insecurity		0.86 (0.60–1.23)	1.02 (0.64–1.61)	1.66 (1.06–2.59) *
Lack of Reliable Transportation		1.31 (0.89–1.95)	1.27 (0.71–2.26)	1.67 (1.03–2.73) *
Utility Insecurity		1.25 (0.86–1.82)	1.99 (1.11–3.58) *	1.92 (1.01–3.65) *
General Health Rating				
General Health Rated as Fair/Poor		1.22 (0.92–1.61)	0.66 (0.38–1.15)	1.07 (0.57–2.00)
15+ Days/Month of Poor Physical Health		1.29 (0.89–1.88)	1.52 (0.86–2.70)	1.64 (0.90–3.01)
15+ Days/Month of Poor Mental Health		1.44 (1.11–1.86) **	1.46 (0.85–2.49)	1.07 (0.65–1.75)
Have Health Insurance		0.87 (0.53–1.46)	1.33 (0.57–3.08)	0.95 (0.45–2.02)

Abbreviations: BRFSS, Behavioral Risk Factor Surveillance System; NH, Non-Hispanic. ^a^ Age groups were based on the BRFSS six-level imputed age variable (_AGE_G): 1 = 18–24 years; 2 = 25–34 years; 3 = 35–44 years; 4 = 45–54 years; 5 = 55–64 years; 6 = ≥65 years. ^b^ Did not Graduate High School, Graduated High School, Graduated from College or Technical School. ^c^ $50,000 to <$100,000, $100,000 to <$200,000, $200,000 or more, Don’t Know/Not Sure/Missing. + *p* < 0.10, * *p* < 0.05, ** *p* < 0.01, *** *p* < 0.001.

## Data Availability

The original contributions presented in this study are included in the article. Further inquiries can be directed to the corresponding author.
